# The mental health of ex-prisoners: analysis of the 2014 English National Survey of Psychiatric Morbidity

**DOI:** 10.1007/s00127-021-02066-0

**Published:** 2021-03-22

**Authors:** Paul E. Bebbington, Sally McManus, Jeremy W. Coid, Richard Garside, Terry Brugha

**Affiliations:** 1 Division of Psychiatry, Faculty of Brain Sciences, University College London, London, UK; 2 National Centre for Social Research, London, UK; 3 Violence and Society Centre, City, University of London, London, UK; 4 Violence Prevention Research Unit, Wolfson Institute of Preventive Medicine, Queen Mary, University of London, London, UK; 5 Centre for Crime and Justice Studies, London, W8 1GB UK; 6 Department of Health Sciences, Leicester University, Leicester, UK

**Keywords:** Ex-prisoners, Epidemiology, National survey, Mental disorders, Social context

## Abstract

**Purpose:**

Prisoners experience extremely high rates of psychiatric disturbance. However, ex-prisoners have never previously been identified in representative population surveys to establish how far this excess persists after release. Our purpose was to provide the first community-based estimate of ex-prisoners’ mental health in England using the data from the 2014 Adult Psychiatric Morbidity Survey (APMS).

**Methods:**

APMS 2014 provides cross-sectional data from a random sample (*N* = 7546) of England’s household population aged 16 or above. Standardised instruments categorised psychiatric disorders and social circumstances. Participants who had been in prison were compared with the rest of the sample.

**Results:**

One participant in seventy had been in prison (1.4%; 95% CI 1.1–1.7; *n* = 103). Ex-prisoners suffered an excess of current psychiatric problems, including common mental disorders (CMDs), psychosis, post-traumatic disorder, substance dependence, and suicide attempts. They were more likely to screen positive for attention-deficit/hyperactivity disorder and autistic traits, to have low verbal IQ, and to lack qualifications. They disclosed higher rates of childhood adversity, including physical and sexual abuse and local authority care. The odds (1.88; 95% CI 1.02–3.47) of CMDs were nearly doubled in ex-prisoners, even after adjusting for trauma and current socioeconomic adversity.

**Conclusions:**

Prison experience is a marker of enduring psychiatric vulnerability, identifying an important target population for intervention and support. Moreover, the psychiatric attributes of ex-prisoners provide the context for recidivism. Without effective liaison between the criminal justice system and mental health services, the vulnerability of ex-prisoners to relapse and to reoffending will continue, with consequent personal and societal costs.

**Supplementary Information:**

The online version contains supplementary material available at 10.1007/s00127-021-02066-0.

## Introduction

### Psychiatric disorder in prisoners

High levels of mental disorder and suicide in prisoners worldwide have long been acknowledged [1–8]. The WHO Regional Office for Europe accordingly initiated a Health in Prisons Programme to address the health needs of people in prisons, with the intention of establishing valid and comparable data. This led to the recent report on the physical and mental health of the 1.5 million people in prison on any given day in the WHO European Region, underlining their coexisting poor mental and physical health, and setting it in the context of “entrenched and intergenerational social disadvantage” [9].

The poor mental health of prisoners has been particularly well documented in Britain. Thus the *National Survey of Psychiatric Morbidity in Prisons* [10] sampled prisoners from every prison in England and Wales. It applied the methods of the first British national survey of psychiatric morbidity [11], thereby allowing direct comparison with the general population. Prisoners experienced an excess of every type of psychiatric morbidity, including common mental disorders (CMD), psychosis, personality disorder, and drug and alcohol problems. At around 10%, the prevalence of psychosis was especially noteworthy [12], and was confirmed using comparable procedures in a substantial prison sample [13]. Similarly, excessive psychiatric morbidity and comorbidity was apparent in a recent psychiatric survey of male and female prisoners in London [14]. Others have noted high levels of PTSD [15].

The WHO report [9] pointed out the adverse consequences of the transition back into the community following release. It also noted that the risk factors for poor health overlap those for incarceration, and bemoaned the lack of continuity of care between health services in prisons and the community. However, little is known of the mental health of prisoners following release. Evidence is confined to recently released or to otherwise unrepresentative samples [16–18].

The high rates of disorder seen in prisoners might arise variously from pre-existing psychiatric morbidity or vulnerability in those who become prisoners, from the psychiatric impact of the prison environment [19], from poor access to effective treatment, and from exacerbated and continuing disadvantage following release. They occur in the context of serious social exclusion, and are associated with increased rates of victimization, both physical and sexual [20]. This in turn has major consequences in the form of suicidal ideation, suicidal behaviour, and non-suicidal self-harm [21].

It has long been accepted that the prison environment is detrimental to the rehabilitation of prisoners with serious mental health problems. In particular, it has proved difficult to organise and deliver adequate psychiatric care in prisons. In one recent study, the treatment needs of nearly half the female prisoners and two-thirds of males were not being met [22]. Thus imprisonment seems virtually guaranteed to exacerbate mental ill-health and to put prisoners at particular risk following release. Lord Bradley's commissioned review of mental health problems and learning disabilities in relation to the English criminal justice system concluded that imprisonment was plainly inappropriate for many offenders with mental disorders and that diversion at various stages of the justice process would be more productive [23].

Recidivism in prisoners is strikingly high: 51% of the people released from custody in England in 2016 received a further custodial sentence within a year [24]. This recidivism seems likely to be linked to the mental health and social situations characteristic of released prisoners. Given the poor recognition and treatment of mental disorders in prisons, the state of prisoners after release should be of particular concern. However, their considerable residential instability makes effective long-term follow-up after release particularly difficult: there has been considerable research on prison populations, but little on former prisoners beyond short-term follow-up studies and analysis of routine data sources (which are generally limited to information on treated conditions and service contacts).

### The current study

The fourth (2014) Adult Psychiatric Morbidity Survey (APMS) [25] was the first to enquire about experiences of imprisonment. It, therefore, provides the first opportunity to measure the size of the ex-prisoner population and to compare the mental health and circumstances of ex-prisoners and never-prisoners in a sample representative of the adult household population of England. We predicted that a history of incarceration would be associated with both psychiatric and social disadvantage, and that social disadvantage would contribute to the mental health problems of ex-prisoners. We accordingly provide detailed accounts of their psychopathological and social characteristics.

Our aims were to:

- Establish the proportion of the community population in England with a history of imprisonment.
- Compare the prevalence of mental disorder and social disadvantage in those with and without such a history.
- Test whether a history of imprisonment is associated with common mental disorder when child and adult adversities and other factors are controlled for.

## Method

### Sampling and procedure

APMS 2014 methods are detailed elsewhere [25]. The survey covered the household population of England aged 16 and above, using a stratified, multistage random sampling design, based on the national Small User Postcode Address File. The final sample comprised 7546 individuals interviewed in their own homes, a response rate of 57%.

The initial phase involved computer-assisted personal interviewing (CAPI), with some sensitive information collected using computer-assisted self-completion interview (CASI), in which the participant used the interviewer’s laptop. They were told beforehand that the interviewer would be unable to see the results of the self-completed parts of the interview. A subset of participants was invited for a second phase interview, during which psychosis and autism were further assessed.

## Measures

### Identifying ex-prisoners

The List of Threatening Experiences (LTE) [26] has formed part of every survey in the series. In the face-to-face interview participants were given a show-card and asked to indicate which, if any, of the listed items they had ever experienced. The items on the show-card are numbered: if they preferred, participants just gave the relevant number to denote endorsement of the experience. The latest survey included an additional item: ‘Spent time in prison on remand or serving a sentence’. No further details of the circumstances of imprisonment were requested.

### Sociodemographic characteristics

The profiles of ex-prisoners and never-prisoners were compared in relation to sex, age, marital/cohabitation status, economic activity, housing tenure, and ethnicity. These were established with standardised survey questions. Due to small numbers, ethnicity was categorised as “white British” or “other”.

### Adverse experiences and circumstances

Experience of abuse in childhood was asked in the self-completion section of the interview. Physical abuse was established with the following question: ‘Not including smacking, before you were 18 did an adult in your life hit, beat, kick, or physically hurt you in any way?’ The experience of non-consensual sex and other forms of non-consensual sexual contact before the age of 16 was similarly established. Service in the Armed Forces at any point was also asked in the self-completion section, after screening for post-traumatic stress disorder (PTSD). Experience of local authority care and homelessness, lack of academic or vocational qualifications, problem debt, and capacity to save (‘Do you (and your family or partner) have enough money to make regular savings of £10 a month or more for rainy days or retirement?’) were asked face to face. Area level deprivation was captured using Index of Multiple Deprivation (IMD) scores, ranked and analysed in quintiles.

### Psychiatric disorders

Non-psychotic psychiatric disorders were assessed in relation to the past week, using the Clinical Interview Schedule Revised (CIS-R) [27]. This provides diagnoses of six common mental disorders (CMDs)—depressive episode, mixed anxiety/depression,^1^ generalized anxiety disorder (GAD), panic disorder, phobic disorder, and obsessive–compulsive disorder (OCD). These are united by central features of affective change, the similarity of their experiential antecedents, and the use of a single instrument to identify them. We, therefore, opted for an overall category of CMD based on the presence in the past week of any of the six types of CMD assessed on the CIS-R. The CIS-R also contains questions about suicidal thoughts (‘Have you ever thought of taking your life, even if you would not really do it?’), suicide attempts (‘Have you ever made an attempt to take your life, by taking an overdose of tablets or in some other way?’), and self-harm without suicidal intent (‘Have you ever deliberately harmed yourself in any way but not with the intention of killing yourself?’). The issues around disaggregating suicide attempts and non-suicidal self-harm are considered elsewhere [28].

Possible cases of current PTSD were identified with the PTSD Checklist (PCL), a 17-item self-report measure covering the DSM-IV criteria for PTSD [29]. A total symptom severity score was derived, ranging from 17 to 85. A positive screen was defined as a score of 50 or more, provided it was accompanied by endorsement of items from each of the three DSM-IV criteria for PTSD (re-experiencing; avoidance and numbing; hyperarousal).

The endorsement of four or more ADHD characteristics on the Adult Self-Report Scale (ASRS) in the phase-one interview was taken as an indication that assessment for ADHD was warranted [30]. Possible autistic traits were identified through endorsement of ten or more items on the Autism Spectrum Quotient (AQ20) [31, 32], which was self-completed. Autistic traits are normally distributed in the population, and are a weak predictor of autism identified using a full assessment. While a full assessment was conducted in the survey’s second phase, too few cases were identified for robust inclusion in this analysis. Borderline intellectual functioning (BIF) was defined as a predicted verbal IQ between one and two standard deviations below the mean (70–85) on the National Adult Reading Test (NART) [33].

The module covering antisocial personality disorder (ASPD) from the self-completion Structured Clinical Interview for DSM-IV Personality Disorders (SCID-II) [35] was completed by participants aged 16–64. A history of criminal behaviour is an identifying feature of ASPD and is thus inevitably present in ex-prisoners.

The procedure for identifying psychosis involved two phases: in the first, participants were screened using the Psychosis Screening Questionnaire (PSQ) [36] together with other criteria indicative of a psychotic episode (such as use of antipsychotic medication, receipt of a psychosis diagnosis, or a stay in a psychiatric ward or hospital). Screen positive individuals were invited for a phase-two assessment, and interviewed by clinically trained research interviewers from the University of Leicester using the Schedules for Clinical Assessment in Neuropsychiatry (SCAN) [37]. In the analyses presented here, we used a measure of *probable psychosis*. This category included SCAN positive cases, together with participants who were not interviewed with SCAN, but who met at least two of the phase-one psychosis screening criteria [38]. As there is evidence that this measure may perform less well in prisoner populations [39], we also analysed endorsement of three or more PSQ items as an alternative: this measure does not include treatment contact as an indicator of psychosis.

### Substance dependence and use

Responses to the Alcohol Use Disorders Identification Test (AUDIT) [40] were used to assess alcohol dependence in the preceding year. A score of 16–19 indicates harmful use/mild dependence, while a score of 20 + identifies probable dependence. Questions about alcohol and drug use were located in the self-completion. Participants who in the past year had used cannabis, amphetamines, crack, cocaine, ecstasy, non-prescribed tranquillisers, heroin, or volatile substances were asked five questions from the Diagnostic Interview Schedule [41] to assess dependence on each drug type. These covered level of use, sense of dependence, inability to abstain, increased tolerance, and withdrawal symptoms. Endorsement of any item in the past year was used to indicate possible drug dependence. For the regression model, a derived variable was used drawing on both the alcohol and drug use measures. We also recorded lifetime use of heroin, and whether participants were currently smokers.

The time frame of the instruments was determined by their standard usage and, therefore, differed between disorders. Thus, CMDs related to the past week, screening for PTSD to the past month, ADHD to the prior 6 months, psychosis and dependence on alcohol and drugs to the past year, while the questions on autistic traits refer to ‘the kind of person you are’ without referencing a specific period.

### Statistical analysis

Our analyses used weighted data and took account of complex survey design, selection probabilities and non-response, rendering results representative of the household population. Population control totals were obtained from the UK Office for National Statistics population estimates for age by sex and region. True (unweighted) sample sizes are presented. The prevalence of psychiatric problems and social circumstances were produced for ex-prisoners and never-prisoners. The significance of differences between groups was established with a *p* value generated through unadjusted binary logistic regressions. Non-overlapping 95% confidence intervals (CI) provided further statistical evidence for differences between groups.

We examined the extent to which the link between incarceration history and common mental disorders can be explained in terms of exposure to adverse contexts and experiences. Logistic regression analyses were run to produce unadjusted and adjusted odds ratios (OR), with CMD as the dependent variable and a history of imprisonment as the independent variable. The adjusted model also included sex, banded age, employment status, housing tenure, debt arrears, area level deprivation quintiles, alcohol problems (grouped AUDIT score), signs of drug dependence, and experience of child abuse. A second model was analysed, with a history of imprisonment as the dependent variable and childhood antecedents and other factors as independent variables. Correlation coefficients were reviewed as a check for collinearity. Missing data were minimal: 36 participants did not respond to the question on prison experience, mostly due to partial completion of the survey. They were excluded from analyses, yielding an analytic sample of 7520. All analyses were conducted in SPSS (version 21.0) or Stata (version 14.1). There were too few female ex-prisoners to consider gender differences in detail.

## Results

Of the 7520 participants, 103 reported having spent at least one period in prison (of whom 19 were women). This gives a weighted population prevalence of 1.4% (95% CI 1.1–1.7). At the time of assessment, 42.5% of ex-prisoners had a CMD, a rate about three times that of the rest of the population (16.6%) (Table 1). 11.9% met criteria for probable psychosis, compared with 0.9% of those who had never been prisoners. Using a score of three or more on the PSQ as an alternative and more conservative psychosis indicator, the equivalent comparison was 2.3% and 0.4%. A third of ex-prisoners screened positive for ADHD, three times the rate for never-prisoners. While we identified a significant level of autistic traits in about 1% of the total sample, in ex-prisoners this figure was nearly 1 in 20 (4.4%): this excess remained when the analysis was limited to male participants. The 13-fold excess in positive screens for antisocial personality disorder is partly but not wholly accounted for by the inclusion of criminal behaviour in the identifying features.

**Table 1:** Mental health problems in ex-prisoners and never-prisoners 1

Mental health problem	Ex-prisoners (*n* = 103) % (95% CI)	Never-prisoners (*n* = 7417) % (95% CI)	Total (*n* = 7520) % (95% CI)	*p*-value
Any common mental disorder	42.5 (31.9–53.8)	16.6 (15.6–17.6)	17.0 (16.0–18.0)	< 0.001
Probable psychosis	11.9 (6.3–21.5)	0.9 (0.7–1.1)	1.0 (0.8–1.3)	< 0.001
Psychosis Screening Questionnaire (PSQ) score 3 or more	2.3 (0.6–8.2)	0.4 (0.2–0.6)	0.4 (0.3–0.6)	0.010
Positive screen for attention-deficit/ hyperactivity disorder	31.0 (21.7–42.0)	9.4 (8.6–10.3)	9.7 (8.9–10.6)	< 0.001
Positive screen for autistic traits	4.4 (1.1–15.5)	0.8 (0.6–1.1)	0.9 (0.7–1.2)	0.018
Positive screen for antisocial personality disorder	36.9 (24.8–50.8)	2.8 (2.3–3.4)	3.3 (2.8–3.9)	< 0.001
Positive screen for Post-traumatic Stress Disorder	26.0 (16.0–39.3)	4.1 (3.6–4.7)	4.4 (3.8–5.0)	< 0.001
Suicidal ideation ever	51.5 (40.1–62.7)	20.1 (9.0–21.1)	20.5 (19.4–21.6)	< 0.001
Suicide attempt ever	25.6 (17.3–36.1)	6.4 (5.8–7.1)	6.7 (6.1–7.4)	< 0.001
Non-suicidal self-harm ever	20.7 (12.4–32.5)	7.1 (6.5–7.9)	7.3 (6.7–8.0)	< 0.001
Signs of drug dependence	24.8 (14.8–38.5)	2.8 (2.3–3.3)	3.0 (2.6–3.6)	< 0.001
Lifetime experience of heroin use	17.3 (10.1–27.9)	0.8 (0.6–1.1)	1.0 (0.8–1.3)	< 0.001
Alcohol				
- harmful use/mild dependence	4.1 (1.2–13.6)	1.8 (1.5–2.3)	1.8 (1.5–2.3)	0.003
- probable dependence	9.5 (4.5–18.8)	1.1 (0.9–1.5)	1.2 (1.0–1.6)	
Smoke 15+ per day	21.3 (14.0–30.9)	5.9 (5.2–6.6)	6.1 (5.5–6.8)	< 0.001
Intellectual impairment (Verbal IQ <85)	37.3 (26.4–49.6)	16.3 (15.1–17.4)	16.5 (15.4–17.8)	< 0.001
Self-recognised learning impairment	10.5 (4.8-21.3)	4.1 (3.6-4.8)	4.2 (3.7-4.9)	0.023

A quarter of ex-prisoners showed signs of dependence on illicit drugs, half were smokers, and one in ten met the threshold for probable alcohol dependence: rates between 3 and 10 times higher than the rest of the population. One in six had taken heroin, compared with less than 1% of the rest of the population. Half the ex-prisoners had considered suicide, and a quarter had made a suicide attempt—four times the rate of those who had not been in prison.

This mental ill-health occurs in the context of factors that reduce competent coping: ex-prisoners were about twice as likely to have both a low predicted verbal IQ and the absence of qualifications. Indeed, one in ten described themselves as having an intellectual or learning impairment.

Ex-prisoners have a demographic and socioeconomic profile distinct from the rest of the population. They were more likely to be male (*n* = 84; 2.4%, 95% CI 1.9–3.1) than female (*n* = 19, 0.4%; 0.2–0.6). While ex-prisoners spanned the age groups, few were 16–24 (0.3%) or over 75 (0.8%), and they were more likely to be aged 35–44 (4.2%). They were also more likely to be currently single, economically inactive, and living in rented accommodation. The proportion of ex-prisoners and never-prisoners who were not white British was similar, at around a fifth (Table 2).

**Table 2:** Socioeconomic profiles of ex-prisoners and never-prisoners

Total		Ex-prisoners		Never-prisoners		Total		*p*-value
		*n*	% (95% CI)	*n*	% (95% CI)	*n*	%	
		103	1.4 (1.1–1.7)	7417	98.6 (98.3–98.9)	7520	100	
By characteristics								
Sex								
Men		84	86.1 (78.1–91.5)	2967	48.4 (47.0–49.7)	3051	48.9 (47.5–50.2)	< 0.001
Women		19	13.9 (8.5–21.9)	4450	51.6 (50.3–53.0)	4469	51.1 (49.8–52.5)	
Age								
16–34		14	21.1(12.4–33,7)	1576	31.1 (29.7–32.5)	1590	31.0 (29.6–32.4)	0.264
35–54		48	49.0 (37.8–60.2)	2420	33.3 (32.1–34.6)	2468	33.5 (32.3–34.8)	
55–74		35	26.2 (18.4–35.9)	2368	25.6 (24.5–26.6)	2703	25.6 (24.6–26.6)	
75+		6	3.7 (1.5–8.6)	1053	10.0 (9.4–10.7)	1059	10.0 (9.3–10.6)	
Ethnicity								
White British		87	81.2 (70.2–88.9)	6296	80.7 (79.3–82.0)	6383	80.7 (79.3-80.2)	0.908
Other		16	18.8 (11.1–29.8)	1113	19.3 (18.0–20.7)	1129	19.3 (18.0–20.7)	
Marital								
Married/cohabitating		42	54.0 (42.9–64.8)	4086	61.9 (60.5–63.2)	4128	61.8 (60.4–63.1)	0.162
Single		38	33.0 (23.8–43.8)	1542	24.2 (22.9–25.5)	1580	24.3 (23.0–25.6)	
Divorced/widowed/ separated		23	12.9 (8.3–19.7)	1789	14.0 (13.2–14.7)	1812	13.9(13.2–14.7)	
Economic activity								
Employed		33	38.9 (28.0–50.9)	3961	59.9 (58.6–61.2)	3994	59.6 (58.3–60.9)	< 0.001
Unemployed		5	3.9 (1.5–9.9)	213	3.4 (2.9–3.9)	218	3.4 (2.9–3.9)	
Economically inactive		65	57.2 (45.7–68.0)	3243	36.7 (35.5–38.0)	3308	37.0 (35.8–38.3)	
Tenure								
Owner occupied		29	29.2 (20.1–40.3)	4892	64.5 (63.1–65.8)	4921	64.0 (62.6–65.3)	< 0.001
Social landlord		51	47.3 (36.0–8.8)	1218	15.5 (14.5–16.6)	1269	16.0 (15.0–17.1)	
Private landlord		23	23.5 (15.1–34.7)	1279	20.0 (18.7–21.3)	1302	20.0 ()	

Significance of the association between characteristic and whether been a prisoner. Weighted analyses; true (unweighted) sample sizes presented

Adverse and distressing life experiences were particularly common in ex-prisoners (Table 3; supplementary Table S1). They were six times more likely to screen positively for PTSD. In addition, they were nearly four times as likely as never-prisoners to report having been physically beaten in childhood by an adult, six times as likely to have been raped in childhood and more than twice as likely to have experienced contact sexual abuse. These bad experiences may be linked to the fact that they were six times more likely to have spent time in local authority care, although we do not know whether the experiences contributed to their being taken into care or occurred while they were in care. Forty percent had a history of homelessness, ten times the rate in the rest of the population. It is also noteworthy that they were twice as likely to have served in the armed forces. Rates of economic inactivity were high, as were financial strain. One in six of the overall sample agreed they would be unable to save £10 a month; in ex-prisoners this rose to 45%.

**Table 3:** Exposure to adverse circumstances in ex-prisoners and never-prisoners to adverse circumstances in ex-prisoners and never-prisoners

	Ex-prisoners % (95% CI)	Never-prisoners % (95% CI)	Total % (95% CI)	*P*-value
*Childhood adversity*				
Childhood beating by an adult	39.9 (28.5–52.4)	11.8 (10.9–12.7)	12.2 (11.3–13.1)	< 0.001
Penetrative sexual abuse before 16	11.1 (5.0–22.9)	1.9 (1.6–2.3)	2.0 (1.7–2.4)	< 0.001
Other contact sexual abuse before 16	17.7 (10.0–9.5)	7.4 (6.8–8.1)	7.6 (6.9–8.2)	< 0.001
Local authority care before 16	11.5 (6.5–19.6)	1.7 (1.4–2.0)	1.8 (1.5–2.1)	< 0.001
*Other adverse circumstances*				
No qualifications	44.4 (33.3–56.1)	20.0 (18.9–21.0)	20.3 (19.3–21.4)	0.014
Lifetime history of homelessness	39.8 (29.0–51.6)	3.4 (2.9–3.9)	3.9 (3.4–4.4)	< 0.001
Served in armed forces	13.6 (7.2–24.2)	6.3 (5.8–7.0)	6.4 (5.9–7.1)	< 0.001
Can’t save £10 a month	45.2 (34.1–56.8)	17.2 (16.2–18.3)	17.6 (16.6–18.7)	< 0.001

Significance of the association between each adversity and whether been a prisoner

People who had been in prison had odds of common mental disorders nearly four times that of never-prisoners in unadjusted analyses (odds ratio (OR) 3.71, 95% CI 2.35, 5.87, *p* < 0.001). This might plausibly be a consequence of their greater exposure to early adversity and extreme social disadvantage. Table 4 presents an analysis controlling for a range of disadvantageous circumstances. While this reduced the specific association between a history of imprisonment and the presence of CMDs, the association remained significant (adjusted OR 1.88. *p* = 0.042). Thus incarceration and the experience of release appear to have appreciable long-term psychiatric consequences.

**Table 4:** Correlates of current common mental disorders: unadjusted and adjusted odds ratios (OR)

	Unadjusted OR (95% CI)	*p* value	Adjusted OR (95% CI)	*p* value
History of imprisonment	3.71 (2.35–5.87)	< 0.001	1.88 (1.02–3.47)	0.042
Female sex	1.72 (1.48–2.00)	< 0.001	1.88 (1.59–2.23)	< 0.001
Age in 10-year bands^a^				
25–34	1.01 (0.77–1.33)	0.944	1.11 (0.81–1.53)	0.496
35–44	1.03 (0.79–1.34)	0.835	1.21 (0.89–1.65)	0.229
45–54	1.01 (0.79–1.30)	0.932	1.26 (0.91–1.73)	0.160
55–64	0.94 (0.72–1.22)	0.638	0.99 (0.72–1.35)	0.928
65–74	0.56 (0.41–0.76)	< 0.001	0.48 (0.34–0.68)	< 0.001
75+	0.41 (0.30–0.56)	< 0.001	0.35 (0.24–0.51)	< 0.001
Employment status^b^				
Unemployed	1.96 (1.37–2.81)	< 0.001	1.43 (0.94–2.18)	0.092
Economically inactive	1.48 (1.28–1.70)	< 0.001	1.97 (1.63–2.39)	< 0.001
Tenure^c^				
Social landlord	2.62 (2.21–3.11)	< 0.001	1.37 (1.11–1.69)	0.004
Private landlord	1.58 (1.33–1.88)	< 0.001	1.05 (0.85–1.30)	0.656
Serious debt problems	4.01 (3.27–4.94)	< 0.001	2.20 (1.71–2.83)	< 0.001
Area level deprivation^d^				
2nd	1.32 (1.03–1.69)	0.028	1.31 (1.01–1.70)	0.039
3rd	1.58 (1.26–1.98)	< 0.001	1.42 (1.11–1.81)	0.005
4th	2.13 (1.66-2.74)	< 0.001	1.72 (1.30-2.27)	< 0.001
Most deprived	2.62 (2.06–3.34)	< 0.001	1.66 (1.26–2.17)	< 0.001
Substance misuse^e^				
Hazardous alcohol use	1.09 (0.88–1.33)	0.431	1.24 (1.00–1.54)	0.055
Drug or alcohol dependence	3.04 (2.38–3.87)	< 0.001< 0.001	2.53 (1.90–3.36)	< 0.001
Abused physically or sexually in childhood	2.97 (2.53–3.48)	< 0.001	2.44 (2.05–2.90)	< 0.001

^a^Reference: 16-24 years

^b^Reference: employed

^c^Reference: owner occupied

^d^Index of Multiple Deprivation quintiles. Reference: least deprived

^e^Reference: no/low risk

## Discussion

### Significance of findings

Although Hawks et al. [42] reported on the notably poor mental and physical health of people on probation in the US, the current study is the first to analyse the psychiatric correlates of a history of imprisonment in a random survey sample of a national population. Our best estimate of the prevalence of ex-prisoners in England equates to approximately 625,000 adults. They had extremely high rates across the whole spectrum of mental disorder, including common mental disorders, psychosis, ADHD traits, and autistic traits. These were accompanied by markedly increased risks of suicidal behaviour. This is consistent with an English study of prisoners in the year following release showing an eightfold excess of suicide in men and a 36-fold excess in women [43]. Imprisonment and mental disorder share social antecedents, in particular childhood maltreatment, abuse and trauma, as indicated by physical and sexual abuse, and living apart from their family of origin [44]. This reflects a population of individuals highly damaged since childhood, with multiple, entrenched social, mental health and substance abuse problems. They tended to have low educational achievement, and a higher prevalence of developmental disorders. This adds to their problems, clearly making it hard for them to acquire, and persist in, employment. They often lived in rented accommodation in areas of concentrated poverty and deprivation. Their mental and physical ill-health and their environmental disadvantage seem likely to exacerbate each other, in a manner suggestive of a *syndemic* [45].

Multiple diagnosis is particularly frequent in prisoners [15], and particularly difficult to manage effectively. Likewise, residential instability after release is common, and makes it more probable that ex-prisoners will fall through the net of supporting psychiatric and probation services. Inadequate or inconsistent material support is likely to increase vulnerability both to mental health problems and to reoffending. Release is thus a point of particular risk, demanding effective liaison between prison mental health services and the community services assuming responsibility after release, as emphasised in England by the influential Bradley report [23].

The poor mental health and social disadvantage of ex-prisoners has implications for their clinical management. The first of these relates to the organisation of release. If the complex social and psychiatric problems faced by ex-prisoners are to be managed effectively, good liaison is necessary, both within and between agencies. This is best served when prison management, probation services, and the psychiatric teams in the prison and the community work together, in tandem with primary medical care, and local authority social and housing services, as required. Done effectively, this will mitigate both mental health difficulties and the risk of recidivism.

However, such liaison has proved extremely difficult. Prison mental health teams should have a leading role in the management of release, and so face the organisational problems of cooperation with multiple community psychiatric services [46]. Communication and collaboration between them and their community counterparts are often complicated by geographical separation, while over-worked community mental health teams may stigmatise ex-prisoners. Release from prison is a point of maximum organisational vulnerability when pre-planned arrangements are particularly likely to unravel, added to which release dates may be changed at short notice [46]. Finally, sudden inter-prison transfers may disrupt the best-laid plans.

Hopkin et al. [46] reviewed interventions targeting this period, finding evidence that some do improve contact with community mental health and other health services, but that the potential impact on reoffending and re-incarceration is complex and further evaluations are needed. Thus, one study of current prisoners reported that one in four had been in contact with mental health services in the year before imprisonment [18].

However, in most jurisdictions, the mental health management of ex-prisoners sooner or later (usually sooner) becomes the exclusive preserve of mental health teams. The problem of course is that ex-prisoners bring their medical, social, and legal predicaments with them following release, while societal failure to deal with these difficulties maintains their vulnerability to relapse and to reoffending, with consequent costs to the offender, to mental health services, and to society.

The second clinical implication relates to the revelation of a history of imprisonment in people referred de novo to mental health services. This is an indication that there may be high levels of vulnerability and need, requiring alert and effective social and psychiatric management.

Community mental health teams, therefore, require to remain alert to the likelihood of complex interacting difficulties in clients with a history of imprisonment. Dual diagnosis is particularly frequent in prisoners, and particularly difficult to manage effectively. Likewise, residential instability makes it more probable that ex-prisoners will fall through the net of supporting services. Inadequate or inconsistent material support is likely to increase vulnerability both to mental health problems and to reoffending.

### Strengths and limitations

To our knowledge, APMS 2014 is a unique attempt to identify a representative sample of ex-prisoners within a national general population survey sample, and in using structured and well-validated methods for determining the presence and social context of a range of mental disorders and conditions. However, at 103, the number of ex-prisoners was small, thereby limiting subgroup analysis. Note also that the interview did not seek details of ex-prisoners’ time in prison: how long ago it was, how many times and how long they were incarcerated, what sort of crimes they had committed, or the type of prison. These attributes might materially affect the link with psychiatric disorder in individual cases, and should be considered for targeting in future research. However, they seem unlikely to invalidate the overall associations we have demonstrated.

The survey is subject to two significant potential biases: (1) it may have failed to sample ex-prisoners proportionately, due to differences in availability and refusal rates and (2) it may not have identified ex-prisoner status in respondents who were successfully recruited.

The sample was restricted to residents in private households. Given that our ex-prisoner category had more frequently experienced homelessness, under-sampling due to current non-residence in private households seems likely. However, this might preferentially remove those with mental health problems. Ex-prisoners may also be more prone to decline invitations to take part in surveys: again, this may particularly affect those with mental disorders. On balance we think both these tendencies would generally operate to *reduce* the apparent differences between ex-prisoners and the rest of the population.

The enquiry about imprisonment was made in the face-to-face part of the interview, using a show-card. While this procedure may have led to under-reporting, the option of revealing prison experience solely by stating its number on the card would have tended to reduce bias arising from a fear of social stigmatisation. However, non-admission of a history of imprisonment would in any case move ex-prisoners to the control group, again reducing group differences in mental ill-health.

The findings here relate to the experiences of ex-prisoners in a single jurisdiction. Different incarceration and release policies may modify the characteristics of ex-prisoners in other countries. However, similar problems of liaison and management seem likely to exist in most developed economies and to have major implications for the implementation of effective rehabilitation.

## Financial support

This analysis is part of independent research funded by the National Institute for Health Research (NIHR) Public Health Research Consortium (PHRC) Policy Research Programme (PHPEHF50/27). The views expressed in the publication are those of the authors and not necessarily those of the NIHR or the Department of Health and Social Care. PEB acknowledges the support of UCLH NIHR BRC. The project was approved by the National Centre for Social Research ethical review committee. Permission to use the data was provided by NHS Digital.

## Role of the funding source

APMS was commissioned by NHS Digital with funding from the Department of Health and Social Care (DHSC) in England. The current analyses include independent research funded by the National Institute for Health Research (NIHR) Policy Research Programme (PHPEHF50/27). The views expressed in this publication are those of the authors and not necessarily those of the NIHR or the DHSC. The funder had no role in the study design, data collection, data analysis, data interpretation, or writing of this paper.

## Supplementary Information

Below is the link to the electronic supplementary material.

Supplementary file1 (DOCX 17 KB)

## Footnotes

1 Otherwise Common Mental Disorder Not Otherwise Specified: CMD-NOS (34).

## Declarations

### Availability of data and material (data transparency)

The Adult Psychiatric Morbidity Surveys datasets are in the UK Data Service archive. Requests for access to the 2014 dataset should be made to the Data Access Request Service at NHS Digital.

### Code availability (software application or custom code)

Survey data collection was programmed in Blaise, a computer-assisted interviewing (CAI) system and survey processing tool for the Windows operating system. The system is developed by Statistics Netherlands and has been designed for use in official statistics. It is available to National Statistical Institutes and related research institutes. Data management and descriptive analyses were conducted in SPSS (v21.0) and regression analyses were carried out in Stata (v14.1). Analysis syntax is available from the authors on request.
