# Supplementary material for: The mental health of ex-prisoners: analysis of the 2014 English National Survey of Psychiatric Morbidity
Source: Soc Psychiatry Psychiatr Epidemiol. 2021 Mar 22;56(11):2083–93. doi: 10.1007/s00127-021-02066-0 (PMC8519824; doi:10.1007/s00127-021-02066-0)
Supplement: Supplementary file 1 — Supplementary file1 (DOCX 17 KB) [file 127_2021_2066_MOESM1_ESM.docx]

**The mental health of ex-prisoners: analysis of the 2014 English National Survey of Psychiatric Morbidity**

**Supplementary materials**

| Supplementary Table 1: Antecedents to imprisonment: unadjusted and adjusted odds ratios (OR) | | | | | | |
| --- | --- | --- | --- | --- | --- | --- |
|  | **Unadjusted OR** | **95% CI** | **P value** | **Adjusted (OR)** | **95% CI** | **P value** |
| Female sex | 0.15 | 0.09-0.26 | <0.001 | 0.12 | 0.06-0.22 | <0.001 |
| Age group^1^  25-34  35-44  45-54  55-64  65-74  75+ | \| 5.51  8.93  6.09  4.53  6.00  1.85 \| \| --- \| | \| 1.18-25.79  2.08-38.37  1.38-26.99  1.01-20.23  1.37-26.31  0.32-10.88 \| \| --- \| | \| 0.030  0.003  0.017  0.048  0.018  0.493 \| \| --- \| | 5.05  6.32  5.00  2.40  5.41  1.32 | 1.06 -24.1  1.40-28.0  1.10-22.8  0.50-11.67  1.20-24.33  0.19-9.22 | 0.043  0.015  0.038  0.276  0.028  0.779 |
| Emotional abuse before age 16 | 4.90 | 2.88-8.34 | <0.001 | 2.58 | 1.47-4.52 | 0.001 |
| Contact sexual abuse before age 16 | 2.59 | 1.31-5.11 | 0.006 | 1.82 | 0.89-3.71 | 0.100 |
| Physical abuse before age 16 | 4.96 | 2.97-8.29 | <0.001 | 2.13 | 1.27-3.57 | 0.004 |
| Experience of local authority care before age 16 | 7.69 | 4.01-14.76 | <0.001 | 3.48 | 1.33-9.15 | 0.011 |

^1^reference 16-24 years
